# Effects of implementing a standardized protocol for identifying and treating iron‐deficiency anemia in pregnancy

**DOI:** 10.1002/pmf2.70089

**Published:** 2025-09-08

**Authors:** Alicia M. Mccarthy, Kimberly Lee, Jeanne A. Darbinian, Justine Chang

**Affiliations:** ^1^ Department of Obstetrics and Gynecology, Kaiser Permanente Walnut Creek Medical Center Walnut Creek California USA; ^2^ Department of Obstetrics and Gynecology, Kaiser Permanente Santa Clara Medical Center California USA; ^3^ Division of Research Kaiser Permanente Northern California Pleasanton California USA

**Keywords:** iron deficiency, iron‐deficiency anemia, iron infusion, pregnancy, standardized protocol, transfusion

## Abstract

**Introduction:**

Iron‐deficiency anemia is a common finding in pregnancy; however, current obstetric practice lacks standardized recommendations for its diagnosis and treatment during pregnancy, and data are limited regarding the effectiveness of diagnosis and treatment strategies toward improving pregnancy outcomes.

**Methods:**

We conducted a retrospective study to examine the impact of a standardized protocol for the diagnosis and management of iron‐deficiency anemia in pregnancy implemented in June 2018 across all 61 prenatal care facilities in an integrated healthcare delivery system. Our primary aim was to compare the prevalence of anemia at the time of delivery among pregnant patients who received prenatal care before protocol implementation with those who received care after protocol implementation. Secondary outcomes included receipt of iron infusions and peripartum red blood cell transfusions. Relative risks for each outcome were adjusted (aRR) for potential confounders.

**Results:**

During the specified pre‐protocol study period, there were 16,151 eligible pregnancies with iron‐deficiency anemia (prevalence 19.5%); during the post‐protocol period, 8604 pregnancies were identified with iron‐deficiency anemia (prevalence 23.3%). Patients with iron‐deficiency anemia receiving prenatal care post‐protocol had 21% lower risk of anemia at the time of delivery compared with those receiving care pre‐protocol (aRR 0.79, 95% confidence interval, CI 0.76–0.83). In stratified analyses, post‐protocol (vs. pre‐protocol) prenatal care was also associated with lower risk of anemia at delivery in each racial and ethnic subgroup. Parenteral iron therapy utilization was more than twofold higher in the post‐protocol cohort (aRR 2.25, 95% CI 2.09–2.42). While not statistically significant, a modest reduction in risk of peripartum blood transfusion was also observed in the post‐protocol cohort (aRR 0.89, 95% CI 0.78–1.02).

**Conclusion:**

Implementation of a standardized protocol for the diagnosis and management of iron‐deficiency anemia during pregnancy was associated with uniformly lower risk of anemia at delivery. Maternal care in the post‐protocol time frame was associated with greater utilization of iron infusions for management of iron‐deficiency anemia and reduced peripartum red blood cell transfusion.

## 1 INTRODUCTION

Anemia in pregnancy, defined by the World Health Organization (WHO) as hemoglobin level < 11 g/dL [1], is a common condition affecting ∼38% of pregnant persons globally and 18%–22% of pregnant persons in high‐income regions. Iron deficiency is the most common etiology [2]. Iron deficiency may exist prior to pregnancy (e.g., due to menstruation‐related losses) and may develop during pregnancy due to increased requirements related to physiologic changes of pregnancy, including expanded maternal red blood cell (RBC) mass and fetal and placental demands. Iron deficiency is a precursor to iron‐deficiency anemia (IDA). In the United States, iron deficiency (with or without anemia) affects up to 30% of third‐trimester pregnancies [3]. Data are limited regarding the prevalence of IDA [4]. Antepartum anemia is associated with an increased risk of cesarean delivery (CD), preeclampsia, postpartum anxiety and depression, postpartum anemia, and peripartum blood transfusion [5, 6, 7, 8]. Anemia is associated with an increased risk of maternal mortality [9, 10, 11]. For the fetus, maternal anemia increases risks of prematurity, neonatal low‐birthweight, low 5‐min Apgar score, and perinatal death [7, 12, 13, 14, 15]. A growing body of literature suggests that iron deficiency during pregnancy precludes appropriate fetal iron loading, and subsequent neonatal iron deficiency is associated with neurocognitive functional differences [16, 17, 18, 19].

The cornerstones of IDA treatment are identifying the root cause of the IDA, and then providing iron supplementation via oral or intravenous (IV) routes. Numerous studies demonstrate the effectiveness of iron supplementation in reducing anemia [20]. Nonetheless, current obstetric practice lacks evidence‐based protocols for diagnosing and managing IDA in pregnancy [4, 21].

Recognizing the combined burden of IDA in pregnancy and the absence of clear clinical management guidelines to address this condition, our integrated healthcare system developed a protocol to standardize the diagnosis and treatment of IDA in pregnancy. The quality goal, through standardization, was to address this common pregnancy condition, both aggressively and equitably. As a result, the Controlling Anemia in Pregnancy (CAP) protocol was introduced into our healthcare system's clinical practice in June 2018 across all maternity centers. Herein, we set out to retrospectively evaluate the effectiveness of the CAP protocol in diagnosing and treating IDA in pregnancy. We hypothesized that implementing a standardized protocol would increase recognition and treatment of IDA during pregnancy. Our primary objective was to compare the prevalence of anemia at the time of delivery among patients diagnosed with IDA during pregnancy who received prenatal care in pre‐CAP compared to post‐CAP time periods. Our secondary objective was to evaluate pre‐CAP versus post‐CAP utilization of IV iron therapy and RBC transfusion.

## 2 MATERIALS AND METHODS

This retrospective cohort study was approved by the organization's Institutional Review Board with a waiver of informed consent. The study used data from a source population of pregnant persons who received prenatal care and delivered a liveborn neonate within our healthcare system. The CAP protocol was initiated in June 2018, and deliveries were ascertained in the pre‐CAP (June 1, 2016 to May 31, 2018) or the post‐CAP (March 1, 2019 to December 31, 2019) period. To avoid including pregnancies that received prenatal care overlapping both periods, the post‐CAP period began 9 months after CAP protocol implementation. Pre‐ and post‐CAP study cohorts were comprised of pregnancies with antepartum anemia *plus* iron deficiency, with deliveries occurring during designated time periods.

**FIGURE 1:**
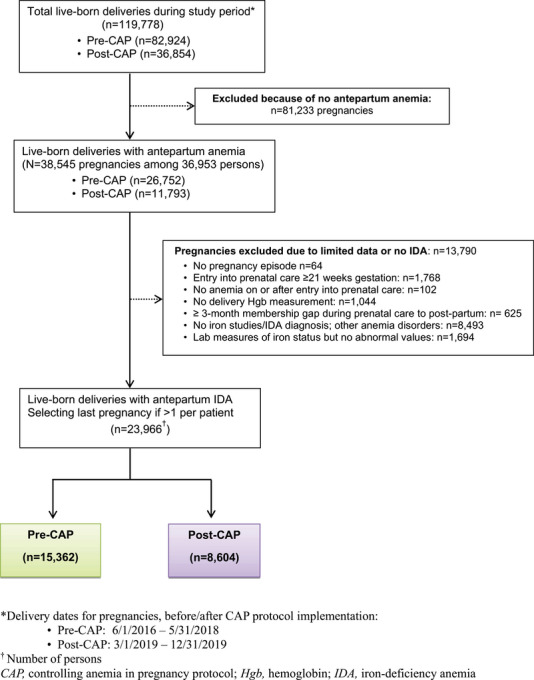
Study cohort selection based on antepartum iron‐deficiency anemia.

Anemia was defined by WHO criteria as hemoglobin < 11 g/dL [2]. Antepartum anemia was defined by having at least one hemoglobin result < 11 g/dL detected on any complete blood count assessment drawn between entry into prenatal care and the period of delivery. The period of delivery was defined as the immediate 96 h prior to fetal delivery. Anemia at delivery was defined by hemoglobin < 11 g/dL obtained during the period of delivery. (If more than one hemoglobin < 11 g/dL was observed during this 96‐h period, the earliest hemoglobin measurement was used.) Otherwise, the phrase “at delivery” refers to the day and time of fetal delivery. Timing of complete blood count assessments reflected routine prenatal care and is recommended on entry to prenatal care, during the mid‐gestation (24–32 weeks), during the period of delivery, and additionally as clinically indicated. During the pre‐CAP period, laboratory evaluation for iron deficiency was not a standard diagnostic recommendation, and iron studies were not included in recommended routine laboratory tests in pregnancy [22]. To reflect clinical practice, we therefore accepted as inclusion criteria the clinical diagnosis of IDA. As such, in pregnancies with established antepartum anemia, iron deficiency was ascertained by either laboratory criteria or by diagnosis code criteria in the absence of laboratory iron studies, further described herein. Laboratory criteria were defined as one or more abnormal iron study laboratory results (Table S1), including a ferritin level below 40 µg/L and transferrin saturation below 20% [23, 24]. Diagnosis code criteria (66.3% of the pre‐CAP cohort, 28.4% of the post‐CAP cohort) included at least one International Classification of Diseases 9th or 10th revision, Clinical Modification (ICD‐9‐CM or ICD‐10‐CM) codes for anemia in pregnancy (ICD‐9‐CM 648.2, ICD‐10‐CM O90.81, O99.0) or IDA (ICD‐9‐CM 280.x, ICD‐10‐CM D50.x) during the designated antepartum time frame, provided there was no evidence of diagnostic codes for other conditions that could impact maternal hemoglobin levels (Table S2). Pregnancies were excluded from analysis if there were antepartum laboratory studies confirming the absence of iron deficiency (or lack of iron‐deficiency diagnoses among those with anemia and no iron studies), lack of hemoglobin measurement during the period of delivery, or incomplete prenatal care within our healthcare system (Figure 1). For individuals with more than one pregnancy during the study period, the last qualifying pregnancy was included.

**FIGURE 2:**
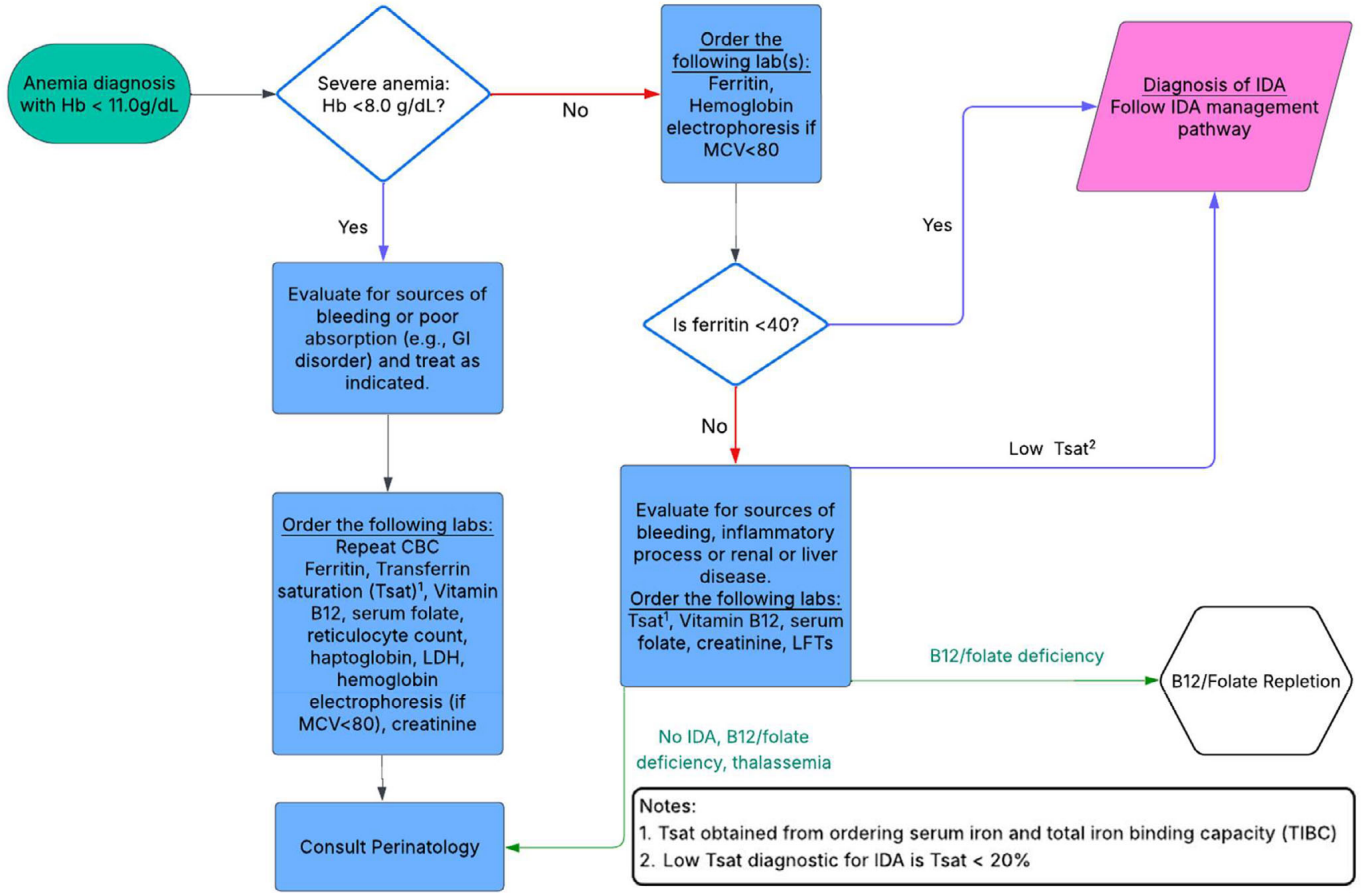
Controlling Anemia in Pregnancy (CAP) protocol—prenatal anemia: initial evaluation. ACOG recommends the use of a ferritin level below 30 ug/L as diagnostic for iron deficiency [4]. The British Society of Haematology also encourages the use of a serum ferritin < 30ug/L as diagnostic for iron deficiency, but also indicates that “there is ongoing debate as to which serum ferritin level to use as threshold to diagnose iron deficiency” and that “it may be appropriate to use a higher cut‐off,” but there are currently insufficient data for this approach in pregnancy [21]. Our regional medical group's Anemia Task Force reviewed the available evidence including that by Guyatt et al. [24] and concluded that using a ferritin level < 40 ug/L is clinically appropriate when considering the prevalence of iron deficiency in pregnancy and that there is a low likelihood of harm when treating with supplemental oral iron. Patients with hemoglobin < 8.0 g/dL are also evaluated for alternate etiologies of anemia concomitantly when testing for iron deficiency.

**FIGURE 3:**
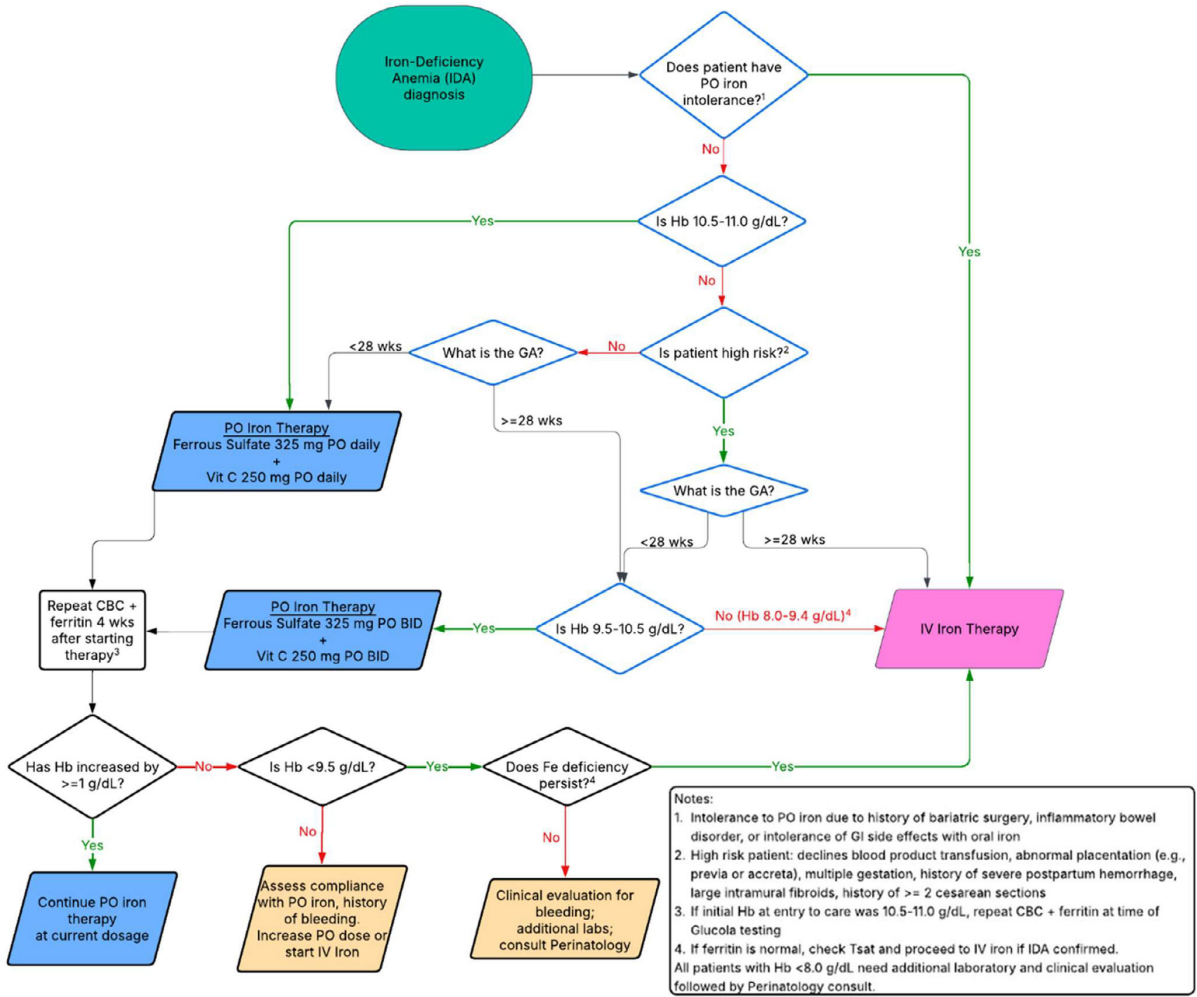
CAP protocol—management of prenatal iron‐deficiency anemia. At advanced gestational age ≥28 weeks, the CAP protocol identifies severe anemia at a higher hemoglobin threshold (any hemoglobin < 9.5 g/dL) to ensure timely IV iron therapy. Following the study period, and based on data in non‐pregnant persons [25], we updated the algorithm and currently recommend oral iron every‐other‐day in all populations when oral iron is indicated.

**FIGURE 4:**
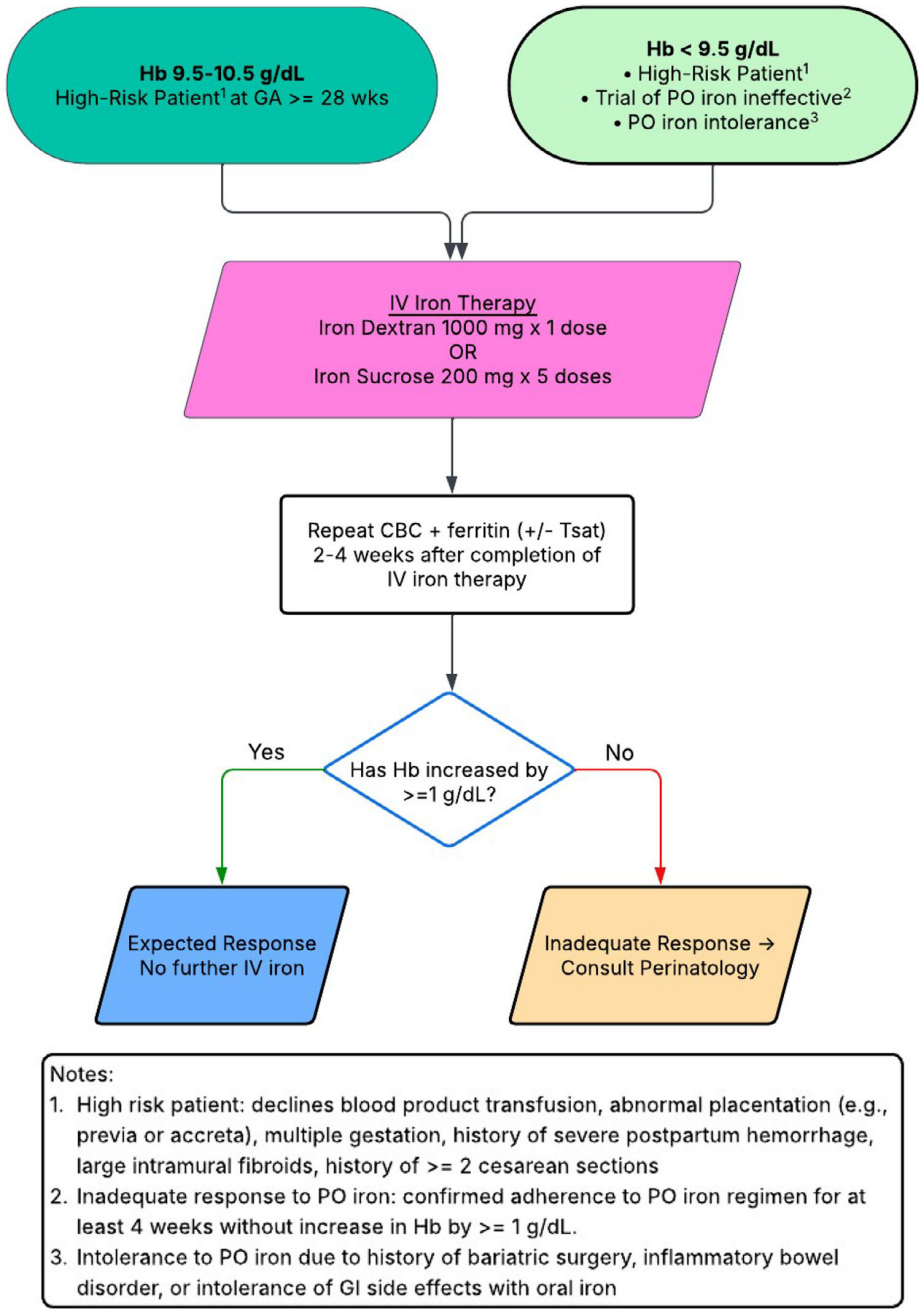
CAP protocol—indications for intravenous iron therapy.

The CAP protocol was developed by a multidisciplinary clinician team within our healthcare system, with the aim of uniformly improving pregnancy outcomes related to anemia. The CAP protocol specifies recommendations for (1) standardizing the evaluation and diagnosis of IDA among identified pregnant persons with antepartum anemia, (2) treatment of IDA with oral and/or IV iron therapy, and (3) follow‐up testing to evaluate adequacy of treatment (Figures 2, 3, 4). Implementation of the CAP protocol utilized a multimodal and multipronged approach including educational resources and presentations for obstetric practitioners; formation of a core group of 37 physician champions in total, who received formal CAP protocol education, met monthly to bi‐monthly surrounding the protocol implementation, and served as expert consultants to their outpatient colleagues at each clinical center; electronic health record systems support through order sets and reflex laboratory orders; and real‐time data tracking with site‐specific feedback. Protocol implementation occurred simultaneously across all 61 outpatient maternity care sites in June 2018.

The primary study outcome was maternal anemia at the time of delivery. The magnitude of change in hemoglobin from the antepartum nadir to delivery was also evaluated. Secondary outcomes included peripartum RBC transfusion (defined as any RBC transfusion between 5 days prior to delivery through 30 days post‐delivery) and IV iron usage defined as receipt of any IV iron between the estimated last menstrual period date (calculated from infant date of birth and recorded gestational age at delivery) and time of delivery. Covariates included maternal age at delivery, self‐reported race and ethnicity, and body mass index based on data recorded at entry into prenatal care for the ascertained pregnancy. Neighborhood socioeconomic status (SES) was estimated using US Census block group level data, where low socioeconomic status was defined as ≥20% of households with income below the Federal poverty designation and/or ≥25% with educational attainment below high school completion within the block group [26]. Clinical characteristics that may have influenced the likelihood of IV iron therapy were ascertained using relevant diagnosis codes linked to clinical encounters or medical problem lists (e.g., history of bariatric surgery or inflammatory bowel disease may contribute to iron malabsorption or intolerance to oral iron where IV iron therapy may be required). Clinical risk factors associated with higher risk of postpartum hemorrhage (e.g., multiple gestation, placenta previa, or concern for placenta accreta spectrum) were also evaluated. Postpartum hemorrhage itself was not included as a covariate in peripartum RBC transfusion analysis since it is in the causal pathway.

Demographic and clinical characteristics, as well as primary and secondary outcomes, were compared using Chi‐square tests for categorical data and *t*‐tests and non‐parametric measures (Wilcoxon–Mann–Whitney test) for continuous variables. All tests were two‐sided, and *p* values < 0.05 were considered statistically significant. For the primary outcome of anemia at delivery, multivariable modified Poisson regression with robust variance was used to estimate relative risk (RR) with 95% confidence intervals (CI) for post‐CAP versus pre‐CAP groups. Characteristics identified as statistically significant in bivariate analyses or deemed clinically relevant were considered as potential covariates in the multivariable models. Between pre‐ and post‐CAP cohorts, analysis of covariance was performed to model the adjusted difference in mean percent change in hemoglobin from antepartum nadir to delivery. The secondary outcomes of peripartum RBC transfusion and IV iron administration during pregnancy were analyzed using modified Poisson regression models. All analyses were performed using Statistical Analysis Software (SAS) version 9.4 (SAS Institute, Inc., Cary, NC).

## 3 RESULTS

During the pre‐CAP study period (June 2016 to May 2018), there were 82,924 pregnancies with live‐born deliveries, of which 26,752 (32.3%) had antepartum anemia. Among pre‐CAP pregnancies with antepartum anemia, 16,151 (60.3%) were classified with IDA based on laboratory (33.8%) or diagnosis code (66.3%) criteria. After restricting to the last pregnancy per person, the final pre‐CAP cohort consisted of 15,362 pregnancies (Figure 1). The prevalence of antepartum IDA among all pregnancies in the pre‐CAP period was 19.5%. During the post‐CAP study period (March to December 2019), there were 36,854 live‐born deliveries, of which 11,793 (32.0%) had antepartum anemia. Among post‐CAP pregnancies with antepartum anemia, 8604 (73.0%) were classified with IDA based on laboratory (71.6%) or diagnosis code (28.4%) criteria, comprising the post‐CAP cohort (Figure 1). The proportion with IDA diagnosed by laboratory studies was more than double that of pre‐CAP patients (33.6%, *p* < 0.001). The prevalence of antepartum IDA among all pregnancies during the post‐CAP period was 23.3%.

Baseline maternal characteristics of pre‐CAP and post‐CAP cohorts with maternal IDA are summarized in Table 1. Patients in the post‐CAP cohort were slightly more likely to have advanced maternal age (AMA), obesity, and vaginal delivery compared to those in the pre‐CAP cohort. The cohorts were similar regarding race and ethnicity, neighborhood SES, parity, gestational age, preterm birth, small‐for‐gestational‐age infant, and prior CD. They were also similar with respect to characteristics that may influence IV iron therapy, including multiple gestation, prevalence of placental disorders (including placenta accreta spectrum), history of bariatric surgery, and history of inflammatory bowel disease.

**TABLE 1:** Cohort characteristics by pre‐ and post‐implementation of the CAP protocol.

	Study group		
Characteristic	Pre‐CAP *N* = 15,362	Post‐CAP *N* = 8604	*p* value^a^
Maternal age at delivery (years)	30.7 ± 5.7^b^	31.0 ± 5.5^b^	< 0.001^c^
Maternal age group			< 0.001
15–24	2317 (15.1%)	1136 (13.2%)	
25–29	3864 (25.1%)	2128 (24.7%)	
30–34	5161 (33.6%)	2936 (34.1%)	
≥ 35	4020 (26.2%)	2404 (27.9%)	0.003
Race/Ethnicity			0.13
Non‐Hispanic White	4498 (29.3%)	2413 (28.1%)	
Black	1708 (11.1%)	957 (11.1%)	
Asian/PI	4146 (27.0%)	2424 (28.2%)	
Hispanic	4379 (28.5%)	2430 (28.2%)	
Other/unknown	631 (4.1%)	380 (4.4%)	
Neighborhood SES (based on US Census block group level)			
Low neighborhood SES^d^	4291 (27.9%)	2420 (28.1%)	0.11
Body mass index (kg/m^2^)	26.9 ± 6.5 ^b^	27.3 ± 6.6 ^b^	< 0.001^c^
Body mass index category			0.002
< 18.5	418 (2.7%)	219 (2.5%)	
18.5 to < 25.0	6794 (44.2%)	3617 (42.0%)	
25.0 to < 30.0	4177 (27.2%)	2355 (27.4%)	
≥30.0	3822 (24.9%)	2328 (27.1%)	< 0.001
Missing	151 (1.0%)	85 (1.0%)	
Delivery mode			< 0.01
Cesarean section	4713 (30.7%)	2455 (28.5%)	< 0.001
Spontaneous vaginal	9913 (64.5%)	5747 (66.8%)	
Assisted vaginal	736 (4.8%)	402 (4.7%)	
Maternal parity (prior to delivery)			0.05
0	6528 (42.5%)	3680 (42.8%)	
1	5183 (33.7%)	2934 (34.1%)	
2	2285 (14.9%)	1315 (15.3%)	
≥3	1366 (8.9%)	675 (7.8%)	
Multiple gestation pregnancy	423 (2.8%)	217 (2.5%)	0.29
Prior cesarean section delivery	2831 (18.4%)	1532 (17.8%)	0.23
Any placental disorder^e^	675 (4.4%)	407 (4.7%)	0.23
Placenta previa	241 (1.6%)	133 (1.5%)	0.89
Abruptio placentae	199 (1.3%)	118 (1.4%)	0.62
Placenta accreta spectrum^f^	34 (0.2%)	19 (0.2%)	0.99
Other diagnosis of retained placenta	246 (1.6%)	165 (1.9%)	0.07
History of inflammatory bowel disease^g^	259 (1.7%)	143 (1.7%)	0.89
History of bariatric surgery	171 (1.1%)	90 (1.0%)	0.63
Preterm delivery (GA < 37 weeks)	1252 (8.2%)	670 (7.8%)	0.32
Early preterm delivery (GA < 32 weeks)	135 (0.9%)	66 (0.8%)	0.36
Small‐for‐gestational‐age	1146 (7.5%)	600 (7.0%)	0.17
Gestational age (weeks)			
At entry into prenatal care	7.2 ± 3.1^b^	7.1 ± 3.0^b^	< 0.001^c^
At nadir antepartum Hgb < 11 g/dL	26.8 ± 5.7^b^	27.0 ± 5.6^b^	0.04^c^
At delivery ^b^	39.1 ± 1.8	39.1 ± 1.7	0.77^c^
At peripartum (delivery) Hgb	39.0 ± 1.8^b^	39.0 ± 1.8^b^	0.91^c^
Nadir antepartum Hgb < 11 g/dL	10.4 [9.9, 10.7] ^h^	10.4 [9.9, 10.7] ^h^	< 0.01^i^

Abbreviations: CAP, Controlling Anemia in Pregnancy protocol; GA, gestational age; Hgb, hemoglobin; PI, Pacific Islander; SES, socioeconomic status.

^a^ From Chi‐square test unless otherwise specified.

^b^ Mean ± standard deviation.

^c^ From *t*‐test.

^d^ Low SES within block group was defined by ≥ 20% households below the poverty income level and/or ≥ 25% with educational attainment less than high school graduate. Note: < 1% with missing data.

^e^ Any of the following during the index pregnancy: placenta previa, abruptio placentae, placenta accreta, percreta, increta, other diagnosis of retained placental. Note that any of the conditions shown under this broader category are not mutually exclusive among cohort members.

^f^ Any of the following during the index pregnancy: placenta accreta, increta, percreta.

^g^ Crohn's disease and/or ulcerative colitis.

^h^ Median [interquartile range].

^i^ From Wilcoxon 2‐sample non‐parametric test, comparing underlying distributions between the two groups.

**FIGURE 5:**
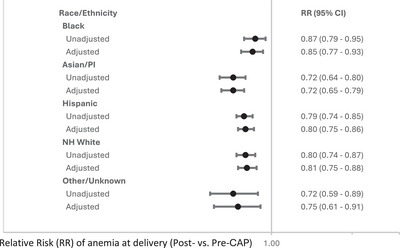
Modified Poisson regression models examining the relative risk (RR) of anemia at delivery associated with implementation of the CAP protocol, stratified by race and ethnicity. CI, confidence interval; PI, Pacific Islander. Unadjusted and multivariable (adjusted for age at delivery, body mass index, parity, prior cesarean section delivery, multiple gestation, and neighborhood socioeconomic status) model results are reported for each race and ethnicity group, comparing the post‐CAP cohort to the pre‐CAP cohort. “Other/Unknown” includes unknown race/ethnicity and persons identifying as multiracial, Native American, or Alaska Native.

Overall, CAP protocol implementation was associated with significantly lower prevalence of anemia at delivery (32.6% vs. 25.6%, aRR 0.79, 95% CI 0.76–0.83) (Table 2a). Furthermore, the significant reduction in anemia prevalence at delivery with CAP protocol implementation was evident across all racial and ethnic groups examined, ranging from 15%–20% reduction among Black, Hispanic, and non‐Hispanic White populations to 28% reduction among the Asian/Pacific Islander (PI) population (Figure 5). Mean percent change between antepartum nadir hemoglobin and delivery hemoglobin level was also significantly higher for the post‐CAP vs. pre‐CAP cohort (Table 2b; mean percent change +14.3% vs. +12.8%, *p* < 0.001).

**TABLE 2:** CAP protocol effect on (a) anemia at delivery and (b) percent change in hemoglobin levels from nadir to delivery.

(a) Anemia (Hgb < 11 g/dL) at delivery		
Outcome	Pre‐CAP *N* = 15,362	Post‐CAP *N* = 8604
Delivery Hgb < 11 g/dL, *n* (%)	5007 (32.6%)	2199 (25.6%)^a^
Relative risk (RR) of delivery Hgb < 11 g/dL with 95% CI		
Unadjusted	Reference	0.78 (0.75–0.82)^b^
Multivariable adjusted	Reference	0.79 (0.76–0.83)^b^, ^c^

(b) Percent change in Hgb between the nadir antepartum value and the measure at delivery		
Outcome	Pre‐CAP (*n* = 15,362)	Post‐CAP (*n* = 8604)
Delivery Hgb (g/dL)	11.4 ± 1.2^d^	11.6 ± 1.1^d^, ^e^
Percent change in Hgb^f^	12.8 ± 11.7^d^	14.3 ± 11.3^d^, ^e^
Difference in mean percent change in Hgb (95% CI)^g^ comparing Post‐CAP to Pre‐CAP		
Unadjusted	1.58 (1.27–1.88)^h^	
Adjusted^i^	1.70 (1.43–1.98)^h^	

Abbreviations: CAP, Controlling Anemia in Pregnancy protocol; Hgb, hemoglobin; RR, relative risk.

^a^ *p* < 0.001 from Chi‐square test.

^b^ *p* < 0.001 from Wald Chi‐square test.

^c^ Adjusted for maternal age at delivery, race and ethnicity, body mass index, parity, prior cesarean section delivery, multiple gestation, socioeconomic status.

^d^ Mean ± SD.

^e^ *p* < 0.001 from T‐test.

^f^ Mean % change between antepartum nadir Hgb measure < 11 g/dL and Hgb measured at delivery; positive value denotes overall increase.

^g^ Linear regression (analysis of covariance) model.

^h^ *p* < 0.001.

^i^ Adjusted for maternal age at delivery, nadir antepartum Hgb < 11 g/dL, race/ethnicity, body mass index in the first trimester of pregnancy, parity, and prior cesarean section delivery.

For the secondary outcome of IV iron utilization, a 2.3‐fold increased use of IV iron was evident in the post‐CAP versus pre‐CAP cohort (crude prevalence: 14.9% vs. 7.0%, aRR: 2.25, 95% CI 2.09–2.42, Table 3). For the secondary outcome of peripartum RBC transfusion (post‐CAP 4.0% vs. 3.4% pre‐CAP), unadjusted analyses showed that the CAP protocol was associated with 14% lower risk of peripartum RBC transfusion (RR 0.86, 95% CI 0.75–0.99, Table 4). After adjusting for maternal age at delivery, race and ethnicity, body mass index, parity, prior CD, multiple gestation, nadir antepartum Hgb < 9 g/dL, any placental disorder, and delivery mode, there was an 11% lower risk of RBC transfusion post‐CAP (aRR 0.89, 95% CI 0.78–1.02) although the difference did not reach statistical significance.

**TABLE 3:** Effect of the CAP protocol on utilization of IV iron therapy during pregnancy.

IV iron received during pregnancy	Pre‐CAP *N* = 15,362	Post‐CAP *N* = 8604	Unadjusted RR (95% CI)	Adjusted RR^b^ (95% CI)
	1073 (7.0%)	1282 (14.9%)^a^	2.13 (1.98–2.30)	2.25 (2.09–2.42)

Abbreviations: CAP, Controlling Anemia in Pregnancy protocol; CI, Confidence Interval; IV, intravenous; RR, relative risk.

^a^ *p *< 0.001 from Chi‐square test.

^b^ Adjusted for maternal age at delivery, race and ethnicity, body mass index, parity, prior cesarean section delivery, multiple gestaton, nadir antepartum Hgb < 9 g/dL, any placental disorder, maternal history of inflammatory bowel disease and bariatric surgery, and socioeconomic status.

**TABLE 4:** Effect of the CAP protocol on RBC transfusion.

RBC transfusion received at delivery^a^	Pre‐CAP *N* = 15,362	Post‐CAP *N* = 8604	Unadjusted RR (95% CI)	Adjusted RR^c^ (95% CI)
	613 (4.0%)	296 (3.4%)^b^	0.86 (0.75–0.99)	0.89 (0.78–1.02)^c^

Abbreviations: CAP, Controlling Anemia in Pregnancy protocol; CI, Confidence Interval; RBC, red blood cell; RR, relative risk.

^a^ Any RBC transfusion received up to 5 days prior to and 30 days after delivery.

^b^ *p *< 0.05 from Chi‐square test.

^c^ Adjusted for maternal age at delivery, race and ethnicity, body mass index, parity, prior cesarean section delivery, multiple gestation, nadir antepartum Hgb < 9 g/dL, any placental disorder, and delivery mode.

## 4 DISCUSSION

In this diverse community‐based patient population encompassing urban and suburban settings, nearly one‐fifth of 82,924 pregnancies were affected by IDA. Implementing a large‐scale, systemwide protocol to uniformly diagnose and treat IDA in pregnancy led to greater recognition of IDA and a significant reduction in anemia prevalence at the time of delivery. This was coupled with a significant post‐implementation increase in mean percent change between antepartum nadir hemoglobin and delivery hemoglobin. Utilization of IV iron also increased. With a lens on maternal outcome, CAP protocol implementation was associated with reduced peripartum RBC transfusions. While adjusted data did not reach statistical significance, we believe the reduction in RBC transfusion is noteworthy and deserves further attention. In total, findings support the implementation of a standardized protocol that addresses the diagnosis and treatment of IDA during pregnancy.

Our results support existing evidence that treating IDA improves maternal hematologic indices [27, 28, 29]. However, in 2015 and again in 2024, the United States Preventive Services Task Force (USPSTF), citing a lack of randomized trials, asserted that there is “limited evidence” on improved maternal clinical outcomes following iron supplementation [30, 31]. Additional studies may change this assertion and bridge an evidence gap that may have dampened enthusiasm for active development and implementation of anemia protocols in pregnancy. Two recent single‐institution quality improvement projects suggest that standardized treatment of iron deficiency in pregnancy may reduce peripartum blood transfusions [32, 33]. Our current report is among the first to demonstrate that a large‐scale effort to standardize the diagnosis and treatment of IDA across > 60 prenatal care facilities was associated with a decreased risk of maternal anemia at delivery.

Our study cohorts were balanced with respect to most patient characteristics except for modest differences in obesity, AMA, and delivery mode. Increasing obesity and AMA in the post‐CAP cohort are consistent with national pregnancy trends over time [34]. The greater proportion of vaginal deliveries in the post‐CAP cohort may be explained by our study period overlap with the California Maternal Quality Care Collaborative (CMQCC), Supporting Vaginal Birth Collaborative. This California‐wide initiative, implemented from 2016 to 2019, aimed to decrease CD rates for nulliparous, term, singleton vertex births; an effect confirmed in an observational study that demonstrated declining CD rates in California during 2014–2019 [29]. Our study also saw a declining proportion of CD in the post‐CAP compared to pre‐CAP time periods. While we adjusted for delivery mode in our RBC analyses, we cannot exclude unmeasured confounding.

Literature remains incomplete in elucidating the full burden of anemia, iron deficiency, and IDA among pregnant persons in the United States, especially data from large healthcare systems. It is notable that the prevalence of anemia and IDA in our pregnant population is higher than that reported in recent publications. Cross‐sectional studies report anemia prevalence in pregnancy as 5.4% [3] and 11.4% [35], and IDA prevalence in pregnancy as 2.6% [36]. Differences in data collection strategies and definitions of anemia and iron deficiency help explain the differences between our patient population and that of prior publications. Our study evaluated anemia and IDA longitudinally through pregnancy. This approach likely reduces the underestimation of anemia during pregnancy, which can occur with cross‐sectional analyses using initial hemoglobin measurement because the first trimester (the evaluation that typically predominates a sampled population [35]) has the lowest prevalence of anemia and iron deficiency. Our study also utilized the WHO definition of anemia [1], whereas prior studies utilized the more restrictive definition of anemia in pregnancy from the Centers for Disease Control and Prevention: hemoglobin < 11.0, < 10.5, or < 11.0 mg/dL in the first, second, or third trimesters, respectively [37]. Furthermore, others defined iron deficiency as ferritin < 12 µg/L, or total body iron < 0 mg/kg [3, 36], whereas our study defined iron deficiency as one or more abnormal iron study results, including ferritin < 40 µg/L (Table S1). Regarding ferritin, our regional medical group's Anemia Task Force reviewed available evidence [23, 24] and concluded that using a ferritin level < 40 ug/L is clinically appropriate when considering the prevalence of iron deficiency in pregnancy and low likelihood of harm with iron treatment.

In line with improved identification of IDA and protocol support of IV iron use in the post‐CAP cohort, our study demonstrates that effective treatment of IDA requires increased IV iron utilization, supporting findings by Flores et al. [32]. While cost differentials between oral and IV iron are important to consider, cost‐effective analysis was not part of this study. A recent cost‐effective model demonstrated cost savings with anemia treatment in pregnancy compared to no treatment and saw the greatest cost savings with IV iron [38].

While iron deficiency is associated with adverse maternal, fetal, and neonatal outcomes, current obstetric practice lacks evidence‐based protocols for the management of IDA in pregnancy. Our study highlights the benefits of an antepartum anemia protocol in reducing the risk of anemia at the time of delivery and introduces a standardized approach for diagnosis and the management of IDA in pregnancy that can be implemented in obstetric practice. Additional studies in large populations and higher‐risk groups are needed to continue evaluating how a successful protocol in reducing IDA in pregnancy can reduce RBC transfusions. Data are also needed to examine how a protocol may improve other maternal outcomes, including postpartum anemia rates, breastfeeding outcomes, and postpartum depression. Research is also needed to further characterize fetal and neonatal benefits of systematic maternal anemia treatment, including birth weight, neonatal iron status, and neurocognitive outcomes. Furthermore, as anemia is an end‐stage manifestation of iron deficiency, research should evaluate whether targeting the identification of iron deficiency in pregnancy reduces the prevalence of anemia and associated risks during pregnancy and the need for IV iron utilization. Indeed, pregnancy can both cause and unmask IDA. Our study is focused on pregnancy risk factors for IDA; pre‐pregnancy factors including heavy menstrual bleeding and pre‐existing iron deficiency were not evaluated. Some guidance implores attention to preconception iron status and iron repletion when indicated, especially in the setting of bleeding disorders [39, 40]. Longitudinal studies are needed to capture the impact of iron‐deficiency diagnosis and treatment throughout the reproductive years, and how pre‐pregnancy iron repletion can mitigate antepartum IDA and its subsequent consequences in pregnancy. A protocol approach to identifying and treating IDA outside pregnancy should also be considered for investigation.

Standardized protocols in medical practice improve patient outcomes and have gained traction in the imperative goal of improving maternal health outcomes in the United States [41, 42]. Protocols improve outcomes by reducing non‐evidence‐based clinical variation. Standardized protocols may also help reduce health disparities. While our study does not comprehensively explore the effects of the CAP protocol on reducing health inequities, it is notable that CAP protocol implementation led to significantly reduced risk of anemia at delivery across all race and ethnicity strata. Protocol creation must take care to exclude practices that could further widen health disparities. For example, at the time of CAP protocol creation, ACOG supported using a lower cut‐off for diagnosing anemia in Black pregnant persons [43, 44]. Our multidisciplinary team rejected these recommendations in favor of creating a protocol without race‐based anemia definitions. Future research is needed to explore the benefits of protocols in general, and anemia‐specific protocols, in reducing health disparities. Our team is currently engaged in such studies.

Strengths of this study include the longitudinal evaluation of hemoglobin and iron studies across pregnancy; and the rigorous, multipronged, system‐wide support for the CAP protocol uptake—a health system‐wide initiative across all outpatient clinics and Labor and Delivery units. The large multihospital medical center population with substantial racial and ethnic diversity increases the generalizability of our findings.

Study limitations include the asymmetry of pre‐CAP and post‐CAP periods examined, but our goal was to examine the implementation of the CAP program prior to the COVID‐19 pandemic. Second, the observational study design precludes conclusions about causation. Observational studies are also prone to biases since they do not involve randomization of the study population. Because we restricted our cohorts to patients with anemia and either laboratory‐confirmed or clinically probable iron deficiency (i.e., anemia by diagnostic code criteria), a higher number of patients with anemia in the pre‐CAP cohort were excluded from analysis since laboratory evaluation for ID was not part of routine pre‐CAP care. This possibly reduced the true magnitude of IDA in the pre‐CAP cohort, blunting the detected impact of the CAP protocol. These selection criteria also led to a higher proportion of pre‐CAP patients with clinically probable IDA compared to post‐CAP patients. Though this created an inclusion‐criteria imbalance between the study populations, we felt this selection strategy was important since it mirrors society guidance, for example, by ACOG, that encourages clinicians to “presume iron deficiency” when anemia is present. While preserving this real‐life practice, we attempted to reduce misclassification bias by excluding patients from analysis if other etiologies for anemia were present (Table S2). Another limitation of observational studies is the potential for unmeasured confounding. Though we attempted thoroughness in accounting for confounders, we cannot rule out that other potential confounders were not evaluated despite our best efforts. Lastly, our study population was restricted to live births; thus, patients with fetal demise and IDA are not captured, and conclusions about this population cannot be made.

## 5 CONCLUSION

Diagnosing and treating anemia in pregnancy is admittedly not a novel management consideration. However, in the absence of a systematic protocol and treatment algorithm, IDA in pregnancy remains underdiagnosed and undertreated, potentially increasing morbidity in pregnancy such as blood transfusion requirements. Our study demonstrates that a standardized protocol for diagnosing and treating IDA in pregnancy was associated with significantly lower prevalence of anemia at delivery across our entire patient population along with reduced peripartum RBC transfusion. Findings encourage the development and adoption of standardized protocols to address IDA in pregnancy.

## CONFLICT OF INTEREST STATEMENT

The authors declare no conflicts of interest.

## FUNDING INFORMATION

Kaiser Permanente Northern California Community Health Program; Kaiser Permanente Northern California Graduate Medical Education Program, Kaiser Foundation Hospitals.

## ETHICS STATEMENT

Institution Review Board exempt project number 1045 was approved on October 2, 2019.

## Supporting information



Supporting information

## Data Availability

Kaiser Permanente Northern California (KPNC) data are not publicly accessible and external access to data is not covered by our KPNC Institutional Review Board–approved study; therefore, there is no uniform resource locator for external access to these data.
