# Supplementary material for: Effects of implementing a standardized protocol for identifying and treating iron‐deficiency anemia in pregnancy
Source: Pregnancy (Hoboken). 2025 Sep 8;1(5):e70089. doi: 10.1002/pmf2.70089 (PMC13344518; doi:10.1002/pmf2.70089)
Supplement: Supplementary file 1 [file PMF2-1-e70089-s001.docx]

**APPENDIX: SUPPLEMENTAL TABLES**

**Supplemental Table 1** - Laboratory criteria for the diagnosis of iron-deficiency anemia

| Laboratory Measure | Qualifying measure |
| --- | --- |
| Ferritin | < 40 µg/L |
| Transferrin saturation | <20% |
| Serum iron | <60 µg/dL |
| Total iron binding capacity (TIBC) | >350 µg/dL |

Our regional medical group’s Anemia Task Force reviewed the available evidence and concluded that it is clinically reasonable to diagnose iron-deficiency using a ferritin level below 40 µg/L and transferrin saturation below 20%.^23,24^ While acknowledging that iron-deficiency cannot be definitively diagnosed based on a serum iron level or TIBC result alone, serum iron levels <60 µg/dL and TIBC levels above 350 µg/dL are observed in iron-deficiency with mild anemia.^44^ These cutoff values were also felt to be appropriate when considering the prevalence of iron-deficiency in pregnancy and the low likelihood of harm with treatment with supplemental oral iron in patients with anemia and these laboratory results.

**Supplemental Table 2** - Alternate anemia diagnoses identified by ICD-9 and ICD-10-CM^a^ codes

| Diagnosis | Codes |
| --- | --- |
| *Hemoglobinopathies* | |
| Sickle cell disease | 282.6x, D57.0, D57.1, D57.2x, D57.8x |
| Thalassemia trait | 282.46, D56.3 |
| Thalassemia major/disease | 282.41, 282.42, 282.43, 282.44, 282.45, 282.47, D57.40, D57.41, D56.0, D56.1, D56.2, D56.5 |
| Thalassemia NOS | 282.40, 282.49, D56.8, D56.9 |
| Other hemoglobinopathies | 282.7, D56.4, D58.2 |
| *Hemolytic anemias – hereditary and acquired* | |
| Hereditary spherocytosis | 282.0, D58.0 |
| Hereditary elliptocytosis | 282.1, D58.1 |
| Anemia secondary to disorder of GSH metabolism | 282.2; D55.0, D55.1 |
| Hemolytic anemia secondary to enzyme deficiency | 282.3, D55.2, D55.3, D55.8, D55.9 |
| Other or unspecified hereditary hemolytic anemias | 282.8, 282.9, D58.8, D58.9 |
| Acquired hemolytic anemias | 283.x, D59.x |
| *Anemias due to nutrient deficiencies* | |
| Vitamin B12 deficiency, including pernicious anemia | 281.0, 281.1, D51.x |
| Folate deficiency | 281.2, D52 |
| Other nutritional anemias |  |
| Other specified megaloblastic anemias not classified elsewhere | 281.3, D53.1 |
| Protein deficiency anemia | 281.4, D53.0 |
| Anemia associated with other specified nutritional deficiency | 281.8, D53.2, D53.8 |
| Unspecified deficiency anemia | 281.9, D53.9 |
| *Other* | |
| Aplastic anemia and other bone marrow failure syndromes | 284.x, D60, D61 |

Patients with any of these diagnoses who did not have antepartum laboratory evidence of iron-deficiency were excluded from the study.

^a^ International Classification of Diseases- 9^th^ revision and 10^th^ Revision, Clinical Modification
